# Berberine protects against diclofenac sodium-induced testicular impairment in mice by its anti-oxidant and anti-apoptotic activities

**DOI:** 10.22038/IJBMS.2022.62811.13895

**Published:** 2022-06

**Authors:** Hanan Waly, Mahmoud Abd-Elkareem, S. A. Raheem, Nasser S. Abou Khalil

**Affiliations:** 1 Laboratory of Physiology, Department of Zoology, Faculty of Sciences, Assiut University, Assiut, Egypt; 2 Department of Cell and Histology, Faculty of Veterinary Medicine, Assiut University, Assiut, Egypt; 3 Department of Pathology, Al Azhar, Faculty of Medicine, Cairo, Egypt; 4 Department of Medical Physiology, Faculty of Medicine, Assiut University, Assiut, Egypt

**Keywords:** Anti-apoptotic, Anti-oxidant, Berberine, Diclofenac, Histology

## Abstract

**Objective(s):**

This study was designed to investigate the effect of berberine (BBR) on diclofenac sodium-induced testicular impairment in mice.

**Materials and Methods:**

Eighteen male mice were divided randomly and equally into three groups for three weeks. One group was kept as control, the second group was injected intraperitoneally with diclofenac sodium (DS) at a dose of 10 mg/kg BW daily during the second and third weeks. The third group received daily oral administration of BBR at a dose of 50 mg/kg BW throughout the whole period of the experiment in parallel with the injection of the above-mentioned dose of DS during the second and third weeks. Plasma testosterone as well as testicular lipid peroxides (LPO), nitric oxide (NO), glutathione (GSH), superoxide dismutase (SOD), and catalase (CAT) were evaluated. In paraffin-embedded testicular tissues, histological examination, immuno-expression of glutathione reductase (GR), and TUNEL assay were carried out.

**Results:**

Testosterone levels were within the normal range in all groups. BBR decreased testicular LPO and induced SOD and GSH without marked changes in CAT and NO. The histology of testis was improved and, regularity and integrity of seminiferous tubules basement membranes, and distribution and amount of peritubular collagen fibers were normalized. BBR treated group showed few positive GR immuno-expression in spermatogenic cells and negative GR immuno-expression in interstitial cells of Leydig along with a few apoptotic spermatogenic cells.

**Conclusion:**

BBR is effective in protecting against DS-induced testicular dysfunction by improving oxidant/anti-oxidant balance and blocking the apoptotic cascade.

## Introduction

Diclofenac sodium (DS) is the most popular consumed nonsteroidal anti-inflammatory medication that has analgesic, anti-inflammatory, and antipyretic properties, and has been shown to be effective in treating a variety of painful and inflammatory conditions (1). Given that testis has a high consumption rate of oxygen, abundance of polyunsaturated fatty acids, and low reserve of anti-oxidant enzymes, whereas DS is a potent inducer of peroxidative damage and programmed cell death (2-4), the testis is considered to be one of the main target organs for DS attack. Nevertheless, administration of DS is associated with a broad spectrum of side effects including nephro-, hepato-, and testicular toxicity (5-7). Among them, testicular toxicity is not fully researched in the literature. DS in rats caused reproductive dysfunction manifested by alteration in semen parameters and testicular histological features along with reduction in testosterone level (7, 8). However, plenty of controversies still emerged about its testicular toxicity relative to strain/age differential response (9) providing a driving force for continuation in exploring this area of research with respect to the multifactorial nature of testicular impairment. DS biotransformation results in overproduction of reactive oxidants which is implicated in cytochrome c release, caspase activation, and DNA fragmentation (10). DS shifted the redox potential towards the pro-oxidant side in rats who suffered from testicular dysfunction by decreasing plasma total anti-oxidant capacity, catalase, superoxide dismutase, and testicular reduced glutathione (7, 8). From the histopathological point of view, DS triggered adverse changes at the levels of seminiferous tubules, and germ, Leydig, and Sertoli cells (7, 11) leading to disruption of androgen biosynthetic activity and sperm supportive nutritive capacity of the testis. 

The search for therapeutic agents, having a protective effect against DS-induced testicular dysfunction with potential natural biological occurrence and predictably having no side effects, is worthwhile. In this regard, berberine (BBR) is regarded as a highly promising strategy owing to its anti-oxidant, anti-apoptotic, and cytoprotective properties (12, 13) giving solid-based rationality to block the multifaceted targets of DS. Therefore, this study aims to investigate the potential protective effect of BBR against DS-induced testicular impairment in mice and its possible mechanistic pathway in a hope of using BBR as a phytochemical in conjunction with DS to reduce its reproductive side effects. 

## Materials and Methods

***Experimental animals and study design***

Eighteen male mice at 5–6 weeks of age and weighing 35 ± 5 grams were obtained from the Animal House, Faculty of Medicine, Assuit University, Assiut, Egypt, and bred under controlled conditions with 12 hr light/dark cycle, temperature of 23 °C, and relative humidity of 55%. Food and water were provided *ad libitum*. After an acclimatization period of one week, mice were randomly and equally divided into three groups, 6 mice each. The experiment lasted for three weeks. One group received no treatment and was kept as a control, while the second group (DS) was injected intraperitoneally with DS at a dose of 10 mg/kg BW (14) daily during the second and third weeks. The third group (DS+BBR) received daily oral administration of BBR (CAS Number: 633-65-8, Sigma-Aldrich Company, USA) at a dose of 50 mg/kg BW (12) along the whole period of the experiment in parallel with injection of the above-mentioned dose of DS during the second and third weeks. 

***Collection and preparation of samples***

After 21 days from the beginning of the experiment, blood samples were collected after overnight fasting from a jugular vein in EDTA-containing tubes. Following centrifuging at 3000 rpm for 10 min, plasma was obtained and stored at -20 °C for estimation of testosterone later on. Mice were killed by cervical dislocation, testes were quickly removed and one testis fixed in 10% neutral buffered formalin for histopathological examination. The other testis was stored at –20 °C to be used for determination of oxidant/anti-oxidant parameters. To prepare 10 % w/v homogenate, 0.1 g of testis was homogenized in 1 ml of 0.1 M phosphate buffer (pH 7.4) using IKA Yellow line DI homogenizer (18 Disperser, Germany). The homogenates were centrifuged at 10000 rpm for 15 min, and the supernatants were kept frozen at -20 °C for subsequent oxidant/anti-oxidant assay.

***Biochemical measurements***

Plasma testosterone level was estimated by ELISA using testosterone enzyme immunoassay test kit (Catalog number: BC-1115) according to the manufacturer’s instruction (BioCheck, Inc., Foster City, USA) with a minimum detectable concentration of 0.05 ng/ml. In the supernatant of testicular homogenate, the total protein level was estimated using Folin phenol reagent following the method of Lowery *et al*. (15). Lipid peroxides (LPO) were measured by thiobarbituric acid reaction according to the procedure of Ohkawa *et al*. (16). Nitric oxide (NO) was measured as nitrite concentration using the method of Ding *et al*. (17). Glutathione (GSH) content was estimated using the method of Beutler *et al*. (18). Superoxide dismutase activity was determined based on its ability to inhibit the autoxidation of epinephrine in an alkaline medium (19). Catalase (CAT) activity was measured according to the method of Lück (20), based on its ability to decompose hydrogen peroxide. All measured parameters were corrected with the total protein levels in the testicular homogenate.

***Histological examination***

The formalin-fixed testes samples were dehydrated in ascending grades of ethanol, cleared in methyl benzoate, and then embedded in paraffin wax. Paraffin sections at 5 µm in thickness were cut and stained with the following histological stains:

1. Haematoxylin and Eosin (HX&E) for general histological examination (21).

2. Periodic acid Schiff (PAS) technique for demonstration of neutral mucopolysaccharides (22).

3. Crossman’s trichrome technique to stain collagen fibers (22).

***Immuno-expression of glutathione reductase***

For immunohistochemical detection of glutathione reductase (GR) in the testis, we used polyclonal anti-superoxide dismutase 2 and anti-glutathione reductase antibodies, respectively (Chongqing Biospes Co., Ltd, China) and Power-Stain™ 1.0 Poly horseradish peroxidase (HRP) DAB Kit (Genemed Biotechnologies, Inc, 458 Carlton Ct. South San Francisco, CA 94080, USA) (23). Sections (5 μm) of paraffin-embedded tissues were dewaxed by immersing the slides in xylene two times for 15 min each; rehydrating the slides in 100%, 100%, 95%, 80%, and 70% solutions of ethanol for 5 min each; and rinsing them in phosphate-buffered saline (PBS) pH 7.4 (3 times for 5 min each). For antigen retrieval, the slides were placed in 10 mM sodium citrate buffer (pH 6.0) and heated to near-boiling (95–98 °C) in a water bath for 20 min. They were then cooled for 20 min at room temperature. The sections were then rinsed in PBS pH 7.4 (3 times for 1 min each). Endogenous peroxidase was inhibited by incubating the slides in 3% hydrogen peroxide for 10 min at room temperature and then washing the slides in PBS pH 7.4 (3 times for 5 min each). The slides were then incubated with the specific primary antibodies (1:100) overnight at room temperature. The slides were then rinsed with PBS pH 7.4 (3 times for 2 min each) and incubated for 15 min in poly HRP conjugate; the slides were then rinsed in PBS pH 7.4 (3 times for 2 min each). Visualization of the bound antibodies was carried out by adding 200 μl of 3,3’-Diaminobenzidine (DAB) substrate solution and incubating the slides for 5–10 min at room temperature. A ready-to-use DAB substrate solution was prepared by adding DAB chromogen solution to DAB buffer solution and then mixing the two solutions in a 1:1 ratio. Then the slides were rinsed with tap water to remove excess substrate solution. The sections were counterstained in Harris hematoxylin for 1 min. The sections were then dehydrated in a graded series of ethanol (95% ethanol and then 100% ethanol), cleared with xylene, and mounted with DPX. 

***TUNEL assay***

Detection and quantification of apoptosis were carried out using an In Situ Cell Death Detection Kit, Fluorescein (Sigma-Aldrich). This TUNEL technology was based on labeling of DNA strand breaks that formed during apoptosis as a result of cleavage of genomic DNA. The damaged DNA emits more fluorescein green color than normal healthy DNA. Sections (3–5 µm) of paraffin-embedded tissues were dewaxed in xylene and rehydrated through a graded series of ethanol and double-distilled water. Then, slides were rinsed in PBS, pH of 7.4 (three times for 5 min each time). The slides were placed in a jar containing 100 ml 0.1 M citrate buffer, pH 6.0, and heated to near boiling (95–98 °C) in a water bath for 30 min followed by cooling for 20 min at room temperature. Sections were then rinsed in PBS at a pH of 7.4 (three times for 1 min each time). TUNEL reaction mixture was prepared by adding the total volume (50 µl) of enzyme solution to 450 µl label solution to obtain 500 µl TUNEL reaction mixtures; then, mixed well to equilibrate components. Slides were rinsed three times with PBS at 15 to 25 °C and excess fluid was drained off. Then drops of TUNEL reaction mixture were added to the samples and slides were incubated overnight in a humidified atmosphere at 37 °C in the dark. Slides were rinsed three times with PBS and directly analyzed under a fluorescence microscope.

All staining preparations were examined by an Olympus BX51 microscope and the photos were taken by an Olympus DP72 camera mounted on the microscope. 

***Statistical analysis***

Data were represented as mean ± standard error of the mean (SEM). The results were analyzed by one-way analysis of variance (ANOVA) followed by Duncan post-test using SPSS program version 16 (SPSS Inc., Chicago, USA). Differences of *P*<0.05 were considered to be statistically significant.

## Results

***Berberine restored the redox balance following diclofenac sodium-induced testicular impairment***

As shown in Table 1, the plasma testosterone level was significantly higher in the DS group than in the control group. DS-induced oxidative stress in the testicular tissue manifested by a significant increase in LPO level and a reduction in GSH and NO levels and SOD activity. However, there was an insignificant change in CAT activity in the DS challenged group versus the control one. 

In the DS+BBR group, plasma testosterone levels decreased but were still significantly higher than in the control level. However, the testosterone level of all groups was within the normal range. BBR succeeded in ameliorating the most hazardous effects of DS on the testicular oxidant/anti-oxidant balance by significantly decreasing the LPO level and increasing SOD activity back towards the control level and by significantly increasing GSH content even above the control level. Nevertheless, the NO level in the DS+BBR group did not significantly change in comparison with the DS group, and CAT activity did not exhibit any significant changes compared with both control and DS groups.

***Berberine improved the histo-architecture of testis following diclofenac sodium challenge***

**Figure 1:**
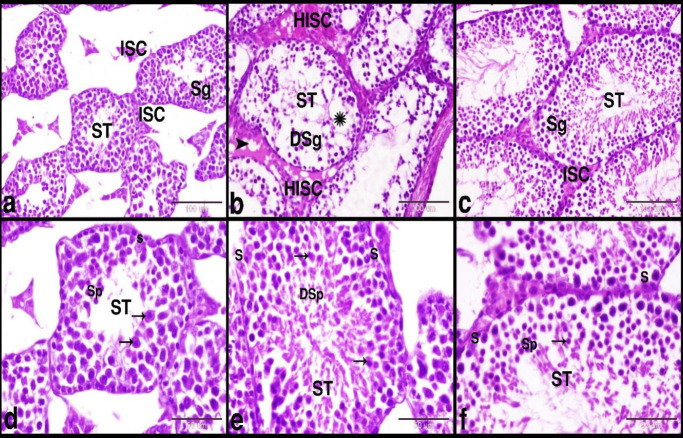
Photomicrograph of paraffin sections showed the protective effect of BBR on DS-induced testicular damage in mice

**Figure 2.**
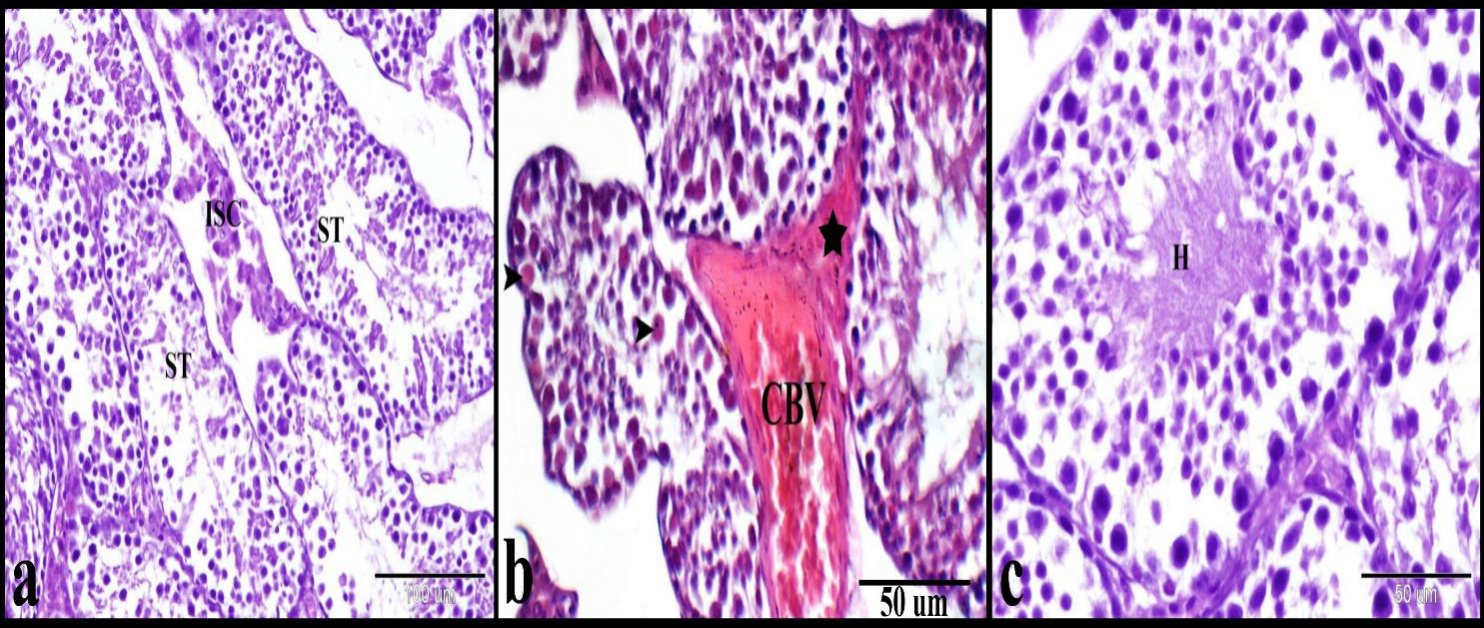
Photomicrograph of paraffin sections showed the DS-induced testicular damage in mice

**Figure 3:**
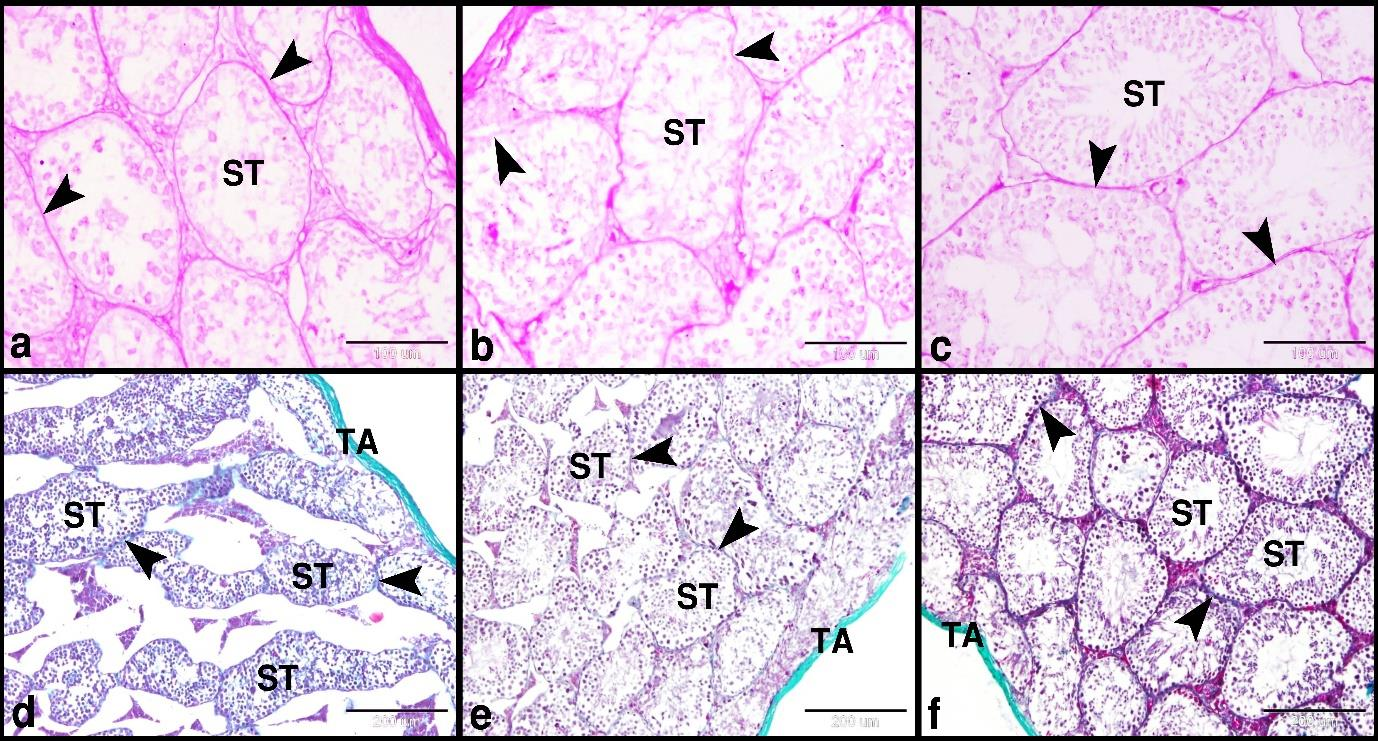
Photomicrograph of paraffin sections showed the protective effect of BBR on DS-induced testicular damage in mice

The microscopical examination of the testes in the control group showed normal histology of the testis which formed of seminiferous tubules separated by numerous interstitial cells of Leydig. Seminiferous tubules were lined by several layers of germinal epithelium (3–7 layers) and Sertoli cells. The stratified germinal epithelium was formed of spermatogenic cells in different stages of maturation (Figures 1a & d). DS group showed distorted and degenerated seminiferous tubules, intratubular and intertubular edema, hyalinized interstitial cells of Leydig, and degenerated spermatogenic cells and spermatids (Figures 1b & e and Figures 2a-c). Whereas the BR treated group showed nearly healthy seminiferous tubules, interstitial cells of Leydig, spermatogenic cells, spermatids, and Sertoli cells (Figures 1c & f). The PAS staining technique was used to evaluate the regularity and integrity of the seminiferous tubules’ basement membranes. The control group showed a regular continued PAS-positive basement membrane of the seminiferous tubules (Figure 3a). While the DS group showed irregular interrupted PAS-positive basement membrane of the seminiferous tubules (Figure 3b). Whereas BBR treated group showed nearly regular and continued PAS-positive basement membrane of the seminiferous tubules (Figure 3c). Crossman’s trichrome technique was used to evaluate the distribution and amount of the peritubular collagen fibers. The Control group showed a normal amount of the peritubular collagen fibers around the seminiferous tubules (Figure 3a). Whereas the DS group showed few, irregular and interrupted peritubular collagen fibers around the seminiferous tubules (Figure 3b). While BBR treated group showed nearly regular and continued peritubular collagen fibers around the seminiferous tubules (Figure 3c).

***Berberine modulated the immuno-expression of glutathione reductase in the testis following diclofenac sodium challenge***

**Figure 4:**
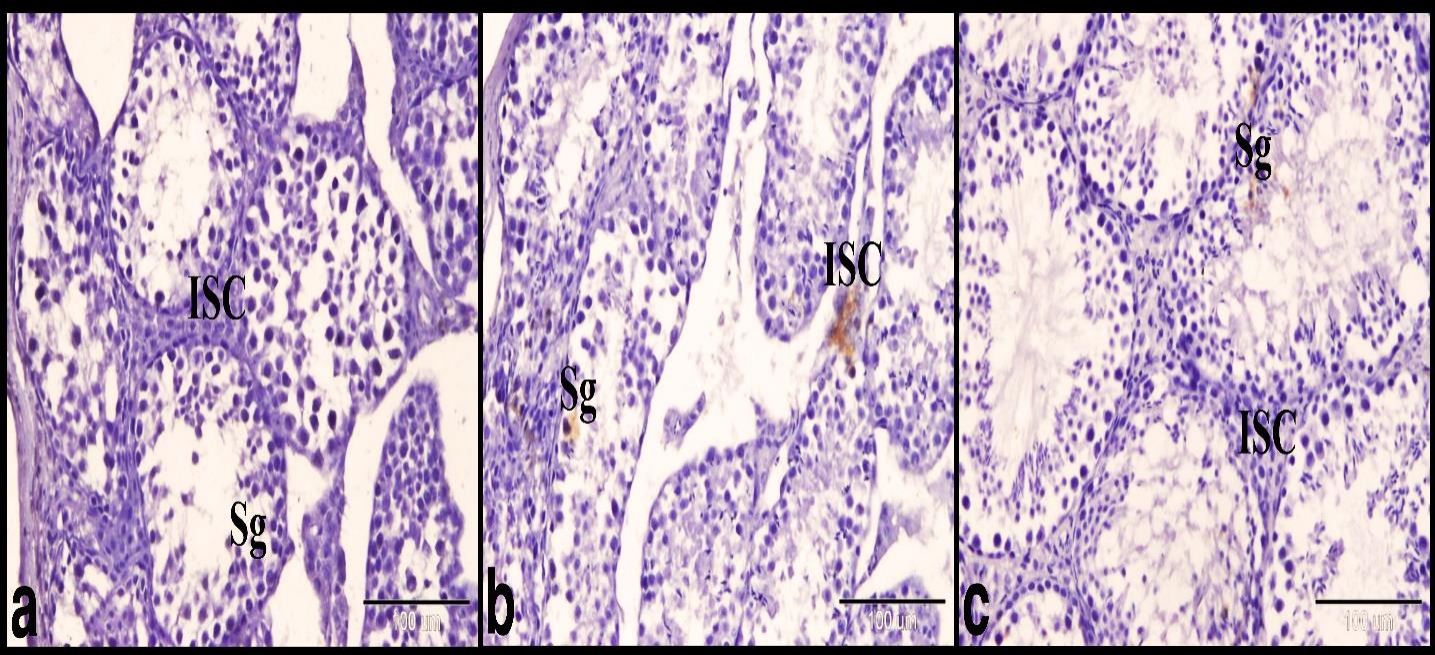
Photomicrograph of GR immunostaining showed the protective effect of BBR on DS-induced testicular damage in mice

Glutathione reductase immunostaining was used to evaluate the oxidative damage in the testes. The control group showed negative GR immuno-expression in the spermatogenic cells and interstitial cells of Leydig (Figure 4a). While the DS group showed few positive GR immuno-expressions in the spermatogenic cells and interstitial cells of Leydig (Figure 4b). Whereas the BBR treated group showed few positive GR immuno-expressions in the spermatogenic cells and negative GR immuno-expressions in the interstitial cells of Leydig (Figure 4c).

***Berberine exerted an anti-apoptotic effect against diclofenac sodium-induced testicular dysfunction***

**Figure 5:**
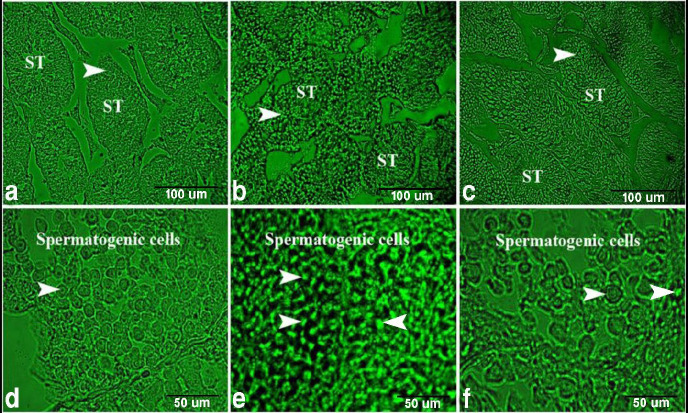
Fluorescent photomicrograph of TUNEL assay in paraffin sections showed the protective effect of BBR on DS-induced testicular damage in mice

For detection of apoptosis, we used a TUNEL assay in paraffin sections. The control group showed few apoptotic spermatogenic cells (Figure 5a & d), but the DS group showed high numbers of apoptotic spermatogenic cells (Figure 5b & e). Whereas the BBR treated group showed few numbers of apoptotic spermatogenic cells (Figure 5c & f) compared with the control.

**Table 1:** Effect of berberine on diclofenac sodium-induced testicular dysfunction in mice

Group	Control	DS	DS+BBR	*P*-value
Parameter				
Testis LPO level (nmol/mg protein)	1.657±0.31	2.212±0.118^a^	1.681±0.061^b^	0.000
Testis NO level (nmol/ mg protein)	9.132±0.838	6.687±0.722^a^	6.316±0.256^c^	0.021
Testis GSH level	36.653±2.843	24.610±1.607^a^	46.103±2.726^bc^	0.000
Testis SOD activity (U/ mg protein)	7.857±0.151	6.244±0.249^a^	8.638±0.635^b^	0.004
Testis CAT activity	1.495±0.416	1.571±0.236	1.751±0.134	0.814
Plasma TAC (mM/l)	0.043±0.005	0.068±0.005^a^	0.048±0.007^b^	0.019
Plasma testosterone level (ng/ml)	2.150±0.764	7.917±0.605^a^	4.850±0.414^bc^	0.000

DS: diclofenac sodium; BBR: berberine; LPO: lipid peroxides; NO: nitric oxide; GSH: reduced glutathione; SOD: superoxide dismutase; CAT: catalase; TAC: total anti-oxidant capacity

Results are expressed as the mean ±SEM of 6 mice per group

a significant difference between DS and control at *P*<0.05

b significant difference between BBR and DS at *P*<0.05

c significant difference between BBR and control at *P*<0.05 (one-way ANOVA followed by Duncan *post hoc *test)

a & d: Control group showed normal histology of the testis; normal seminiferous tubules (ST), interstitial cells of Leydig (ISC), spermatogenic cells (Sg), spermatids (Sp, arrow), and Sertoli cells (S). b & e: DS group showed degenerated seminiferous tubules (ST), intratubular (star) and intertubular (arrowhead) edema, hyalinized interstitial cells of Leydig (HISC), degenerated spermatogenic cells (DSg), degenerated spermatids (DSp, arrow) and Sertoli cells (S). c & f: BR treated group showed nearly healthy seminiferous tubules (ST), interstitial cells of Leydig (ISC), spermatogenic cells (Sg), spermatids (Sp, arrow), and Sertoli cells (S). Original magnification; a, b & c X200, scale bar = 100 μm; d, e & f X400, scale bar = 50 μm, Hematoxylin and Eosin stain

a: DS group showed degenerated seminiferous tubules (ST) and slight hyperplasia in the interstitial cells of Leydig (ISC). b: DS group showed distorted seminiferous tubules with scattered degenerated spermatogenic cells (arrowhead), and mildly edematous interstitium (Star) with markedly dilated congested blood vessel (CBV). c: DS group showed seminiferous tubules with a hyalinized center (H). Original magnification; a X200, scale bar = 100 μm; b & c X400, scale bar = 50 μm, Hematoxylin and Eosin stain

a: Control group showed the regular continued PAS-positive basement membrane (arrowheads) of the seminiferous tubules (ST). b: DS group showed the irregular interrupted PAS-positive basement membrane (arrowhead) of the seminiferous tubules (ST). c: BBR treated group showed nearly regular and continued PAS-positive basement membrane (arrowheads) of the seminiferous tubules (ST). d: Control group showed normal peritubular collagen fibers (arrowhead) around the seminiferous tubules (ST). e: DS group showed few, irregular, and interrupted peritubular collagen fibers (arrowhead) around the seminiferous tubules (ST). f: BBR treated group showed nearly regular and continued peritubular collagen fibers (arrowhead) around the seminiferous tubules (ST). Note the connective tissue of tunica albuginea (TA). Original magnification; a, b & c X200, scale bar = 100 μm, periodic acid-Schiff (PAS); d, e & f X200, scale bar = 100 μm, Crossmon's trichrome technique

a: Control group showed negative GR immuno-expression in the spermatogenic cells (Sg) and interstitial cells of Leydig (ISC). b: DS group showed few positive GR immuno-expression in the spermatogenic cells (Sg) and interstitial cells of Leydig (ISC). c: BBR treated group showed few positive GR immune expressions in the spermatogenic cells (Sg) and negative GR immune expressions in the interstitial cells of Leydig (ISC). Original magnification; X200, scale bar = 100 μm

a & d: Control group showed few apoptotic spermatogenic cells (arrowhead) in the seminiferous tubules (ST). b & e: DS group showed a high number of apoptotic spermatogenic cells (arrowhead) in the seminiferous tubules (ST). c & f: BBR treated group showed few apoptotic spermatogenic cells (arrowhead) in the seminiferous tubules (ST). Original magnification; a, b & c X200, scale bar = 100 μm; d, e & f X400, scale bar = 50 μm

## Discussion

Knowledge regarding the potential testicular disturbances during DS medication will allow for its protection by giving complimentary herbal-based supplements to ameliorate its adverse effects. BBR in this study exhibited anti-oxidant and anti-apoptotic effects against DS administration. 

DS shifted the testicular oxidant/anti-oxidant balance towards the oxidant side similar to that found previously (8). Excessive oxidation of lipids as shown by increased LPO levels under the DS challenge could change the physicochemical characters of cellular membranes resulting in covalent modification of proteins and nucleic acids in the testis (24). This is suggested as a leading cause of deterioration of the testicular histological features in the DS group. By studying the biodegradation pathways of DS, suppression of glutathione conjugation by overdosage causes damage to mitochondrial transmembrane which is implicated in inhibition of the anti-oxidant activity (25). NO exhibits a broad array of regulatory functions in the testis including regulation of local blood flow, germinal cell maturation and apoptosis, Sertoli cell tight junction dynamic, and Leydig cell steroidogenesis in addition to its cytoprotective action (26). Thus, the ability of DS in our experimental model to reduce the testicular NO content contributes to compromising specific testicular functions and alters the reproductive homeostasis. Overproduction of reactive oxygen species under DS attack may promote reversible uncoupling of NO synthase and shift the enzyme activity to produce more reactive oxygen species instead of NO (27, 28) resulting in testicular NO depletion. GSH, a major intracellular anti-oxidant, has a fundamental role in male fertility (29). Depletion of GSH is matched with that found in the testis of rats (8) and in harmony with depletion of glutathione redox system in DS-induced hepato-, nephro- and gastrointestinal toxic rat models (30-32). In the current experiment, testicular SOD activity was suppressed in the DS group consistent with that shown in rats (8) most probably due to down-regulation of SOD protein expression (33). Taking into account that it dismutates the harmful superoxide anion, its deficiency leads to impaired fertilizing ability of sperms (34).

BBR supplementation to the DS group in our study was effective in restoring testicular oxidant/anti-oxidant balance as that found in gossypol-induced testicular toxicity in rats (35), and in agreement with a growing body of evidence emerging from other oxidative stress-induced animal models (36, 37). This indicates the ability of BBR to protect against the peroxidative injury of testicular tissue by a free radical scavenging property. The reduction in intracellular superoxide anion level (38) and up-regulation in SOD expression (39) could be involved in returning SOD activity toward the normal level. Attenuation of endoplasmic reticulum stress (40) and inhibition of aldose reductase and NADPH oxidase activity (41) contribute to normalization of reactive oxygen species generation and subsequent reduction in LPO level following oral administration of BBR. Increased GSH levels may be caused by down-regulation of glutathione peroxidase expression (42). 

However, failure of BBR to induce any significant change in the NO level and CAT activity in this study is in contrast with its well-established anti-oxidant activities and reinforces the rationality behind estimation of the overall oxidative/reductive status of the tissue samples by measuring TAC. Nevertheless, evidence has been amassed from the literature denoting the diversity in the response patterns to BBR supplementation in relation to the differences in the studied doses and experimental models. One of the key secrets responsible for the anti-carcinogenic property of BBR is its ability to stimulate overproduction of reactive oxygen metabolites causing cell death by apoptosis and increased autophagy levels (43). This fact implies the double-faced redox effect of BBR. 

Although there were significant differences between the testosterone levels of the different experimental groups, the testosterone levels were still within the normal range. Failure of DS to induce change in the steroidogenic ability of testis confirms that Leydig cells are relatively resistant to chemotherapy-induced damage (44). For example, Leydig cells remain resistant to death even when exposed to high doses of cadmium (45). 

Although testosterone levels in the DS group exceeded the control levels, disturbance in the process of spermatogenesis appeared obviously based on our histopathological findings. In view of previous literature (7, 46), this finding represents a major surprise. It was suggested that DS induced a biphasic pattern modulation in testosterone secretion and that a potential early reduction in the testosterone level, which was not investigated in our study, could increase secretion of LH through feedback mechanism leading to Leydig cell hyperplasia (47). This indicates the necessity of studying the possible endocrine disturbance by DS across the hypothalamic-pituitary-gonadal axis through a time window to analyze LH receptors on the Leydig cells and the gene expression of steroidogenic enzymes. Leydig cell hyperplasia provides histological evidence of the motivating effect of DS on the testicular steroidogenesis relative to the duration of exposure and the dose of chemotherapy in our experimental design. It was hypothesized that the disturbance in the delicate balance of the cellular redox system could be involved in the induction of Leydig cell proliferation (47). Oxidative stress in our experimental model presumably causes intense expression of phospholipase C, an enzyme involved in cell proliferation, which evokes Leydig cell hyperplasia and induces cholesterol-transport-related enzyme expression contributing to the increase in Leydig cell androgen biosynthesis (48).

The disruptions in the testicular histological features following the DS challenge are in the same line as previous studies (7, 8, 11). The degenerative changes in the germinal cells may be attributed to the reduction in its count and disturbances in the microenvironment of Sertoli cells that provide a suitable morpho-biochemical background needed for attachment and development of the germ cells (11, 49, 50). The appearance of mildly edematous interstitium could be related to the ability of DS to evoke oxidative stress (7, 8) which causes loss of the integrity of the inter-Sertoli tight junction leading to increased permeability of the blood-testis barrier (51). DS has the ability to cause microvasculature injury as that observed in the kidney of prenatally administrated rats (52).

Restoring the testicular histo-architecture by BBR supplementation is matched with its protective effect against other testicular dysfunction models (12, 35). This outcome could be explained based on the fact that BBR promotes germ cell differentiation and spermiogenesis progression *via* protection of cellular DNA from genotoxicity, down-regulation of pro-apoptotic p21 expression, and maintenance of cyclin-D1 and cdk4 gene expression (53). Acceleration of spermatogonial stem cell renewal and maintenance of the Leydig-Sertoli cells network could be the causative factors behind restoring spermatogenesis in the seminiferous tubules (54). According to a previous study (35) and also as evident from the findings of the current study, the anti-oxidant and anti-apoptotic activities of BBR contribute to its cytoprotective effect on the testicular tissues. 

The role of DS in targeting critical key points of the apoptosis pathway could be incriminated in the pro-apoptotic effect of DS on the testis by reactive oxidants-mediated induction of Akt-Bid-Cytochrome c-caspase pathway (10), increased abundance of pro-apoptotic caspase 3 (55), Bcl-2 protein transcript, caspases 3 and 9 activities, and decreased protein level of anti-apoptotic Bax (56). DS induces apoptosis by stimulating reactive oxygen species production and decreasing the mitochondrial membrane potential (57) which results in increased escape of cytochrome c and apoptogenic factors from the mitochondria, and finally triggers the caspase-dependent and caspase-independent apoptosis (58).

Using TUNEL staining, the anti-apoptotic effect of BBR on the testis of the DS group is matched with the middle cerebral artery occlusion model by regulating the expression of apoptosis-related protein leading to inhibition of the mitochondrial-dependent caspase apoptotic pathway (59). Literature is punctuated with emerging evidence from ischemic reperfusion injured models indicating the mechanistic avenues by which BBR excretes anti-apoptotic effects including attenuation of endoplasmic reticulum stress (60) and modulation of PI3K/Akt/mTOR (61) and AMPK signaling pathways (62). 

## Conclusion

The results of the present study illustrated that BBR supplementation improved the redox balance and blocked the apoptotic cascade in the testicular tissue inflicted by DS. Thus, this phytochemical ingredient could be a promising candidate as a supplementary natural agent along with DS protocol to ameliorate its harmful impacts.

## Authors’ Contributions

HW Provided conceptualization and methodology; MA Helped with data curation, methodology, and editing; SAR Provided methodology; NSA Provided statistical analysis, interpretation of the data, and writing. All authors take responsibility for the content of the submitted manuscript. 

## Conflicts of Interest

The authors declare no conflicts of interest.
